# Hippocampal Cortactin Levels are Reduced Following Spatial Working Memory Formation, an Effect Blocked by Chronic Calpain Inhibition

**DOI:** 10.3390/brainsci5020241

**Published:** 2015-06-19

**Authors:** Mikel L. Olson, Anna E. Ingebretson, Katherine M. Harmelink

**Affiliations:** Department of Psychology and Program in Neuroscience, Concordia College, Moorhead, MN 56562, USA; E-Mails: aeingebr@cord.edu (A.E.I.); kjohns12@cord.edu (K.M.H.)

**Keywords:** radial arm maze, hippocampus, spatial working memory, cortactin, calpain

## Abstract

The mechanism by which the hippocampus facilitates declarative memory formation appears to involve, among other things, restructuring of the actin cytoskeleton within neuronal dendrites. One protein involved in this process is cortactin, which is an important link between extracellular signaling and cytoskeletal reorganization. In this paper, we demonstrate that total hippocampal cortactin, as well as Y421-phosphorylated cortactin are transiently reduced following spatial working memory formation in the radial arm maze (RAM). Because cortactin is a substrate of the cysteine protease calpain, we also assessed the effect of chronic calpain inhibition on RAM performance and cortactin expression. Calpain inhibition impaired spatial working memory and blocked the reduction in hippocampal cortactin levels following RAM training. These findings add to a growing body of research implicating cortactin and calpain in hippocampus-dependent memory formation.

## 1. Introduction

A functioning hippocampus is essential for optimal declarative memory formation. Declarative memories that include information about the spatial environment or an organism’s orientation to that environment (spatial memories) are particularly affected by hippocampal dysfunction [1]. Rodents that have had selective hippocampal lesions show marked impairment in spatial memory tasks, both in long-term spatial reference memory tasks such as the circular water maze [2] and in shorter-term spatial working memory tasks such as the radial arm maze (RAM) [3].

The mechanism by which the hippocampus aids in the consolidation of memories is the subject of a sizeable body of research and many molecules which subserve memory formation in the hippocampus have been identified. Of these molecules, those which regulate the structure of dendritic spines may be especially important for ensuring the ongoing stability of a newly formed memory [4]. Indeed, dysregulation of dendritic spine structure has been implicated in many human psychological and neurological disorders that include symptomatic declarative memory impairments [5]. Among the factors that are believed to drive dendritic structural changes in healthy neurons are glutamate signaling and subsequent activation of *N*-Methyl-d-Aspartate (NMDA) receptors, events which are crucial for hippocampus-dependent memory formation [6,7] and long-term potentiation (LTP) [8,9]. The precise mechanism by which glutamatergic activation of NMDA receptors leads to changes in dendritic spine structure is unknown, but it appears to involve the regulation of actin dynamics within the neuron.

One molecule which plays an important role in the formation and stabilization of actin filaments is the F-actin binding protein cortactin. Cortactin has multiple neuronal functions including cell motility, endocytosis, axon guidance, and cytoskeletal remodeling [10,11]. Relevant to its potential role in synaptic plasticity and memory formation, cortactin is concentrated in dendritic spines of hippocampal neurons and is an important regulator of activity-dependent changes in spine morphology, a process which has been shown to be NMDA receptor-dependent [12,13]. Cortactin alters dendritic structure in part due to its ability to bind to both F-actin and the actin nucleating protein Arp2/3 in order to stabilize and expand the actin cytoskeleton [14,15]. Changes in the activity of cortactin can be triggered by multiple and varied events [10], and the activation state of cortactin is highly regulated by a number of tyrosine and serine kinases (for a recent review, see [16]). One of the primary tyrosine phosphorylation sites on cortactin is Y421 [17] and this site is regulated by several non-receptor tyrosine kinases including those in the Src-family [18].

At the behavioral level, only two studies to date have directly examined the potential role of hippocampal cortactin in learning and memory formation. Meighan and colleagues observed a significant decrease in hippocampal cortactin 4 h after spatial memory formation in the water maze, an effect which was blocked by NMDA antagonism [19]. Conversely, Davis and colleagues showed an up-regulation in cortactin 12 h following exposure to a novel environment [20]. Additional research is needed to further elucidate the potential role of cortactin in hippocampus-dependent memory formation. Therefore, in our first experiment, we sought to determine if spatial working memory formation in the RAM leads to reduced total hippocampal cortactin levels. In addition, we tested whether or not Y421-phosphorylated cortactin is altered by RAM training.

Cortactin is degraded by calpains [21], of which calpain 1 and calpain 2 are the most common forms in the central nervous system. Calpains are cysteine proteases that are activated following NMDA receptor activation and subsequent elevations in intracellular calcium [22]. Calpains have many roles within the cell, and inhibition of calpain impacts memory formation. In healthy organisms, chronic calpain inhibition impairs novel object memory and spatial memory [23,24,25]. In addition, several studies have shown that calpain inhibition impairs LTP in hippocampal slices [26,27,28]. Overall, these studies indicate that calpain may play a role in mediating learning and memory processes in the hippocampus. Because cortactin is a proteolytic target of calpain, in our second experiment we tested the hypothesis that calpain inhibition might affect spatial memory formation in the RAM and alter hippocampal cortactin levels.

## 2. Experimental Section

### 2.1. Animals

Male Sprague-Dawley rats (Charles River Laboratory, Wilmington, MA, USA) approximately nine months of age weighing 350–500 g were used in all experiments. Animals were housed in a colony room on a 12 h light/dark cycle, with the light period of the cycle beginning at 07:00. All behavioral training and testing took place during the light period. Five days prior to behavioral training, animals were food-restricted in order to reach a target weight (90% of free-feeding weight). Rats received a minimum of 10 g of food per day, with water available *ad libitum*. Animal care and experimental procedures were carried out in compliance with NIH guidelines and were approved by the Concordia College Institutional Animal Care and Use Committee (Protocol #Mikel_Olson12-05-2011).

### 2.2. Apparatus

An eight-arm radial arm maze (RAM) constructed from opaque Plexiglas was used for all behavioral training and testing. The maze consisted of an octagonal center platform and eight equally-spaced arms extending from the center. A plastic food cup (3 cm in diameter and 1.5 cm deep) was affixed to the floor at the end of each arm. The maze was elevated 0.8 m above the ground in a 2 m × 2.5 m testing room illuminated with fluorescent light. Brightly-colored paper of different shapes was affixed to three walls in the testing room to provide extra-maze cues. The position of the researcher in front of the fourth wall served as an additional cue. Removable blocks were used during the training phase in the RAM to prevent entry into selected arms.

### 2.3. Behavioral Procedure

Prior to behavioral training, animals were exposed to human contact 5 min/day for three days and habituated to the RAM for 10 min/day for two days. During habituation sessions, animals were placed inside the RAM and allowed to explore the maze for 10 min in the presence of a novel food item (Honey Nut Cheerios^®^) scattered throughout the RAM.

Following habituation, animals were trained using the spatial win-shift (SWSh) paradigm in the RAM. The SWSh task assesses spatial working memory, the ability to use recent spatial variables to guide behavior [3]. The SWSh task has been shown to be sensitive to manipulation of the hippocampus but not other limbic structures or the striatum, indicating that task performance is not a result of habitual stimulus-response conditioning [29]. Furthermore, the SWSh task has the advantage over spontaneous alternation protocols, because it can distinguish between delay-dependent and delay-independent spatial working memory impairments [30]. These factors make the SWSh task particularly useful for investigating the neurobiological correlates of hippocampus-dependent memory formation. The details of the SWSh protocol have been reported previously [31,32]. Briefly, the task consisted of a training phase and a testing phase. During the training phase, all eight arms of the RAM were baited with a food item (1/2 Honey Nut Cheerio^®^), but four randomly selected arms were blocked to prevent entry. The combination of blocked arms changed daily, but all animals shared the same combination on a given day. Animals had a maximum of 300 s to locate the food in the four open arms. The training phase ended when the animal had entered the four open arms of the RAM with all four paws or when 300 s had elapsed. Animals were returned to their home cages for a 5 min delay period after the training phase. Immediately following the delay, animals were returned to the RAM for the testing phase. During the testing phase, all eight arms of the RAM were open, but food was only available in arms that had been blocked in the training phase. Optimal test performance was defined as entry into the four arms that contained food, without repeated entries. The testing phase ended when animals had entered all four of the correct arms or when 300 s had elapsed. In the testing phase, errors were defined as entry into any arm that did not contain food and were divided into two types. Across-phase errors were defined as entrance into an arm that was open during the training phase. Within-phase errors were defined as repeated entry into an arm that was blocked during the training phase. The number of each type of error, as well as the time to complete the task, was recorded in the test phase. Animals were trained on the SWSh task for 21–24 days until reaching baseline levels of performance, defined as a group average of less than two across-phase errors and less than one within-phase error over three consecutive days. To determine the probability of animals attaining these criteria by chance, a Monte Carlo simulation was run on 100,000 randomly-behaving rats using MATLAB software (Natick, MA). The likelihood of making an average of three or fewer total errors over three consecutive days by chance was 1.6%. Therefore, this was used as the justification for determining our baseline performance criteria. Animals were excluded from the study during acquisition training if they used a sequential search strategy (choosing arm 1, then 2, then 3, *etc.*) or if they repeatedly failed to explore the RAM (less than 4 choices in 300 s). A total of two animals were excluded from the study based on these criteria. Control animals were placed inside the RAM for roughly an equivalent length of time but were not trained on the SWSh protocol. These animals remained naïve to the SWSh procedure and served as untrained controls for biochemical analysis.

### 2.4. Surgery

Animals were anesthetized with equithesin without chloral hydrate ([33]; 5 mg/kg, i.p.; active ingredient sodium pentobarbital 35 mg/kg; Sigma P-3761) before being mounted on a stereotaxic apparatus (Stoelting, Wood Dale, IL, USA). An intracerebroventricular cannula (Alzet Brain Infusion Kit 2 #0008663, Durect Corporation, Cupertino, CA, USA) was positioned using coordinates −1.0 mm posterior and 1.5 mm lateral from bregma according to a stereotaxic atlas [34]. The cannula was connected via flexible polyvinylchloride catheter tubing (9 cm) to a miniosmotic pump (model # 2002, Durect Corporation, Cupertino, CA, USA). The pumps have an approximate fill volume of 250 μL and continuously deliver 0.5 μL/h over the course of 14 days. Calpain Inhibitor I (Sigma #A-6185) and calpeptin (Santa Cruz Biotechnology #SC-202516) were dissolved in DMSO and diluted in 10% Cremaphor EL/saline to yield a concentration of 3.4 mM calpain I and 1.62 mM calpeptin. These doses are similar to those used by Shimizu *et al.* [23]. Animals in both the drug and vehicle conditions received solutions that had a final concentration of 25% DMSO and 7.5% Cremaphor EL in saline. Prior to surgery, the pumps were prepared, loaded, and primed according to the manufacturer’s instructions. The pumps were then implanted subcutaneously between the scapulae. Placement of the cannula in the lateral ventricle was confirmed by a post-mortem injection of fast-green dye (Sigma #F-7252). No animals were excluded from the study because of incorrect placement.

### 2.5. Immunoblotting

Animals were sacrificed by decapitation immediately following the testing phase of the SWSh task. After extracting the whole brain and placing it on an iced petri dish, the hippocampi were quickly dissected and frozen at −70 °C until homogenization. Hippocampi were homogenized in buffer (20 mM Tris HCl, 137 mM NaCl, 0.1% SDS, 10% glycerol, 1% NP-40 Tergitol, 0.0184% sodium orthovanadate, 2 mM EDTA, 1 μg/mL protease inhibitor cocktail), to yield a final sample concentration of 1 mg wet tissue weight/mL. Homogenates were centrifuged at 12,000 rpm for 10 min at 4 °C. Supernatant samples were further diluted 1:1 with homogenization buffer, mixed with loading buffer, and heated at 100 °C for 5 min. SDS-PAGE was carried out using 10% Tris HCL gels (Bio-Rad). Following electrophoresis, samples were transferred to a nitrocellulose membrane. Membranes were blocked for 1 h in 5% Tris-Buffered Saline (TBS) milk buffer for probing cortactin or 5% TBS filtered BSA buffer for probing phosphorylated cortactin. Primary antibody for total cortactin (mouse anti-cortactin 1:2000, Millipore #05-180) or phosphorylated cortactin (rabbit phospho-specific tyrosine-421 (Y421) anti-cortactin 1:1000, Millipore #AB3852) was added to the buffer, and the membranes were incubated overnight. Following incubation, membranes were rinsed in alternating TBS/ Tween 20 TBS (TTBS) washes and treated with a horseradish peroxidase linked secondary antibody for cortactin (anti-mouse IgG 1:5000, Cell Signaling Technology #7076) or phosphorylated cortactin (anti-rabbit IgG 1:5000, Cell Signaling Technology #7074) and incubated for 1 h. Membranes were rinsed again in alternating TBS/TTBS washes. To visualize protein expression, membranes were treated with a chemiluminescent substrate (SuperSignal^®^ West Pico, Thermo Scientific, IL, USA) for 5 min and exposed to photographic film (Kodak × Omat LS film). Film was developed in a darkroom using Kodak film development chemicals. Amido Black total protein stain (Sigma #A-8181) was used as a loading control and prepared using methodology previously described [35]. This method, while used less frequently as a loading control than α-actinin or glyceraldehyde-3-phosphate dehydrogenase, has been demonstrated to be a reliable alternative to single protein loading controls [35]. The entire lane of each sample was quantified to control for loading variance. Total Lab 100 (Newcastle, UK) was used to quantify the optical density of protein bands. Band densities for cortactin and Y421-phosphorylated cortactin were first converted to percent of total protein by dividing the value of protein immunoreactivity by the value of Amido Black total protein stain. Next, data were expressed as a percentage of untrained controls.

### 2.6. Behavioral Procedure

#### 2.6.1. Experiment 1

Thirty-two animals were used in this study. Following acquisition of baseline performance criteria on the SWSh task, experimental animals were randomly divided into groups and received two additional days of training on the SWSh task, with the inter-phase delay set at 5 min (*n =* 8), 30 min (*n =* 8), or 4 h (*n =* 8). Untrained control (U) animals (*n =* 8) were exposed to the RAM but not trained on the SWSh task. Trained animals were sacrificed immediately after the testing phase of the SWSh task, and untrained animals were sacrificed following habituation to the RAM. Hippocampal brain tissue was removed and analyzed using the immunoblotting procedure outlined above.

#### 2.6.2. Experiment 2

Twenty animals were used in this study. Following acquisition of the SWSh task, animals were randomly divided into a calpain inhibitor group (*n =* 10) and a vehicle group (*n =* 10). Mini-osmotic pumps containing 3.4 mM calpain inhibitor I/1.62 mM calpeptin or vehicle were surgically implanted for chronic ICV drug infusion. Animals were allowed to recover from surgery over five days. Following surgical recovery, animals received six additional days of SWSh training in the RAM with the inter-phase delay set at 30 min. On the final day of behavioral training, animals were sacrificed immediately after the testing phase, and hippocampal tissue was removed and analyzed via immunoblotting.

## 3. Results and Discussion

### 3.1. Task Acquisition

**Figure 1:**
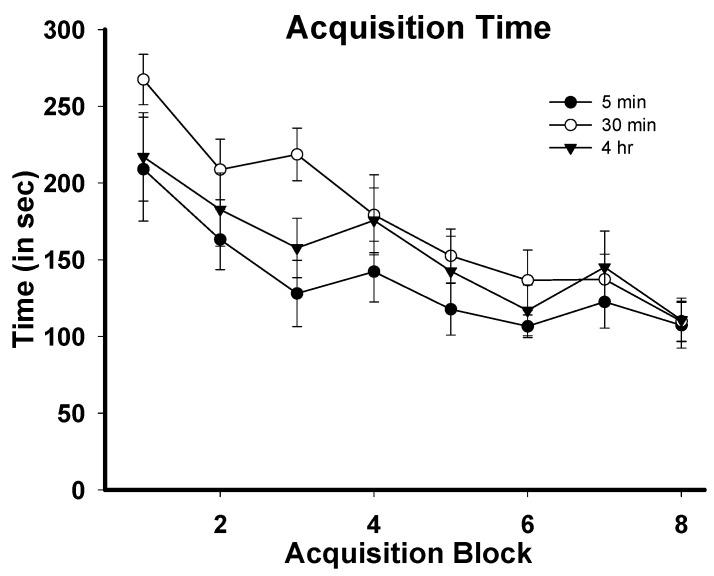
Mean latencies to complete the training phase of the SWSh RAM task during acquisition. Each data point represents the group mean (±SEM) of each experimental group for training block 1–8.

Figure 1 depicts mean latencies to complete the training phase of the SWSh task during task acquisition. Data for each rat’s daily task performance were averaged across two to three-day blocks in order to account for the fact that there were differences in the number of acquisition days required to reach baseline performance (21–24 days). The result of this was that each rat had a score for block 1–8 of acquisition training. The data points in Figure 1 represent group averages for each of these blocks. For latency to complete the task, a 3 (group) × 8 (training block) repeated measures ANOVA was conducted and revealed a significant main effect for training block: F(7,154) = 7.426, *p =* 0.000, no main effect for group F(2,22) = 2.395, *p =* 0.12, and no interaction effect F(14,154) = 1.026, *p =* 0.454. The significant effect for training block indicates that animals in all groups learned the task during the acquisition period. Similarly, for within-phase errors, there was a significant effect for training block F (7,154) = 3.044, *p =* 0.031, no significant effect for group F(2,22) = 2.353, *p =* 0.12, and no significant interaction effect F(14,154) = 0.918, *p =* 0.54. For across-phase errors, there was no significant effect for training block F (7,154) = 1.759, *p =* 0.1, a significant effect for group F (2,22) = 3.956, *p =* 0.034, and no significant interaction effect F (14,154) = 0.514, *p =* 0.922. The significant group effect for across-phase errors was found between the 30 min and 4 h groups (Tukey’s = *p =* 0.039), but not the 5 min group, indicating that the 4 h group took longer than the 30 min group to acquire the task with regard to across-phase errors. Importantly, one-way ANOVAs showed that there were no differences between any of the groups on block 7 or block 8, indicating that there were no group differences in across-phase errors by the end of the acquisition phase.

### 3.2. Total Cortactin and Y421-Phosphorylated Cortactin are Transiently Reduced Following RAM Training

**Figure 2:**
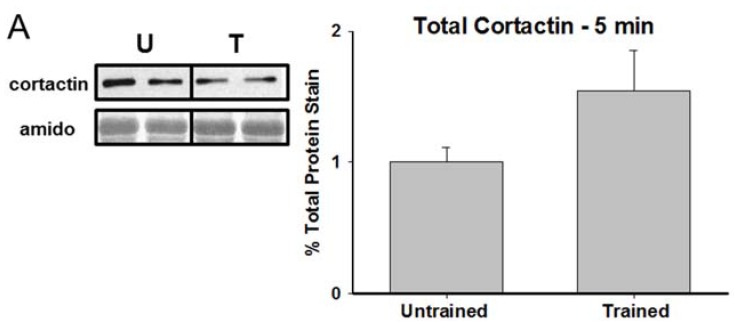

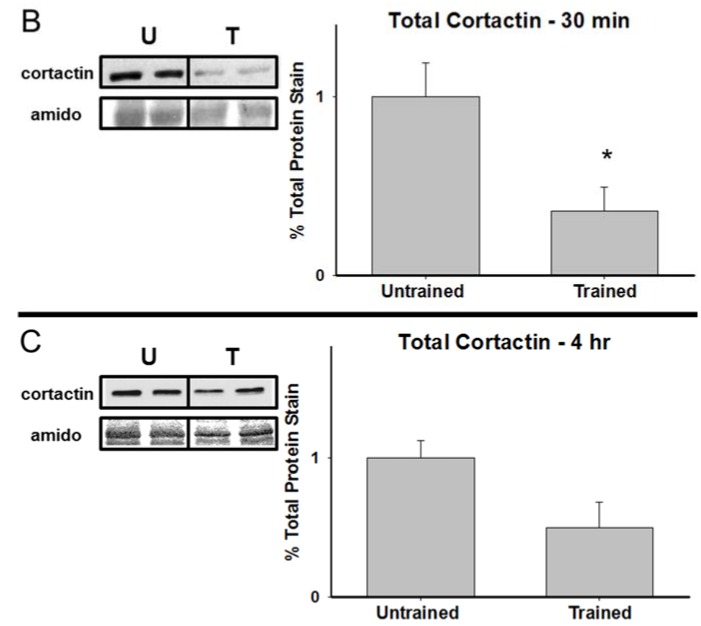
Cortactin is transiently reduced 30 min after RAM training. Representative immunoblots and group means for hippocampal cortactin (±SEM; expressed as percent of untrained controls) at 5 min, 30 min, and 4 h following RAM training. * *p* < 0.05 compared to untrained controls.

**Figure 3:**
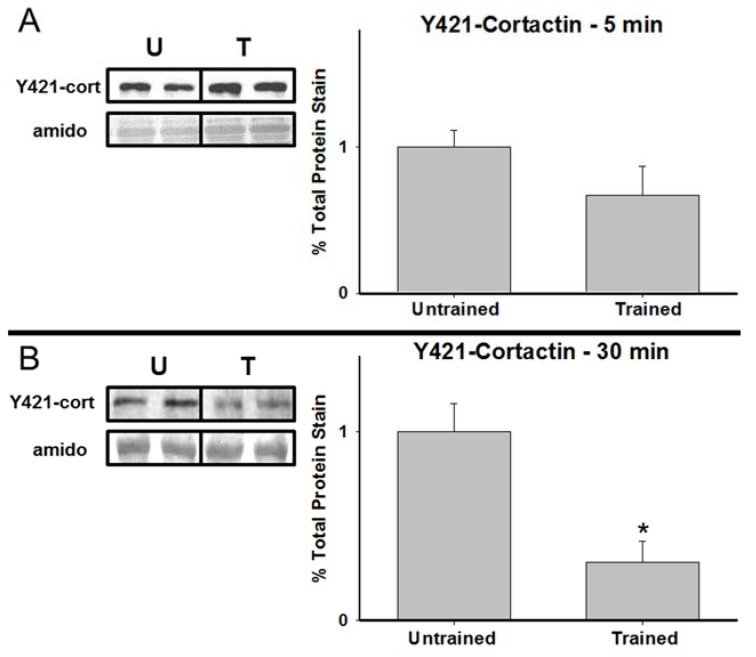

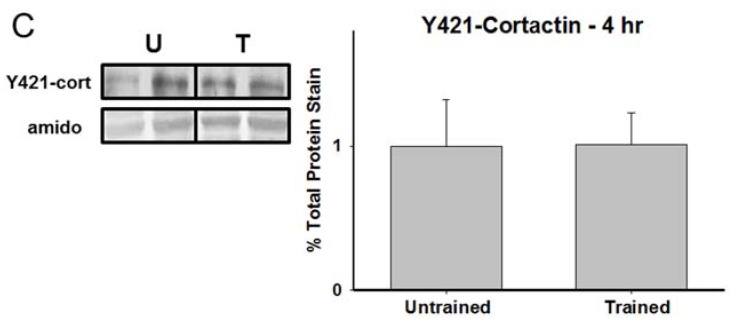
Y421-phosphorylated cortactin is reduced 30 min after RAM training. Representative samples and graphical depictions of group means for hippocampal Y421-phosphorylated cortactin (±SEM; expressed as percent of untrained controls) at 5 min, 30 min, and 4 h following RAM training. * *p* < 0.05 compared to controls.

Figure 2 shows representative immunoblots and graphical representations, expressed as percent of untrained controls, of hippocampal cortactin expression in untrained animals and animals in the 5 min, 30 min, and 4 h experimental groups. While there were no significant differences between the groups at 5 min T(6) = −1.636, *p =* 0.15 and only a marginal difference at 4 h T(6) = 2.276, *p =* 0.07, there was a significant reduction in total cortactin observed at 30 min T(6) = 2.751, *p =* 0.033. Figure 3 presents corresponding data for Y421-phoshphorylated cortactin. Similarly, no significant differences were observed between the untrained group and either the 5 min T(6) = 1.417, *p =* 0.20 or 4 h T(6) = −0.016, *p =* 0.99 time points. However, as with the total cortactin data, there was a significant reduction in Y421-phosphorylated cortactin in the hippocampus of the 30 min group T(6) = 3.678, *p =* 0.01.

### 3.3. Chronic Calpain Inhibition Impairs Spatial Working Memory Formation on the SWSh Version of the RAM

**Figure 4:**
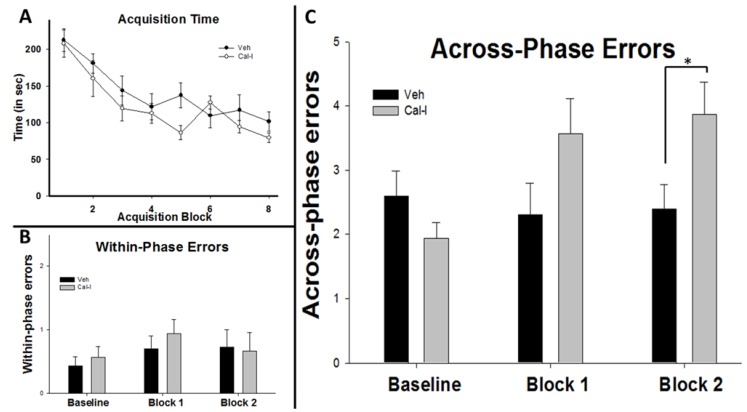
Chronic calpain inhibition impairs spatial working memory formation in the RAM. Animals in both groups acquired the task (**A**) with both groups showing pre-surgery latencies that were not significantly different overall or on block 7 or block 8. Post-surgery, there were no differences between Veh and Cal-I groups for within phase errors (**B**); but a significant increase in across-phase errors for the Cal-I group was seen (**C**) compared to Veh controls. * *p* < 0.05.

In a separate experiment, additional animals were first trained to baseline performance, then surgically implanted with osmotic pumps and catheters which delivered chronic intracerebroventricular (icv) injections of either vehicle (Veh) or calpain inhibitors (Cal-I). Regarding pre-surgical acquisition training, for time to complete the task, a 2 (group) × 8 (training block) repeated measures ANOVA revealed a significant main effect for training block F(7,126) = 20.84, *p =* 0.001. This indicates that prior to osmotic mini-pump implantation, animals in both the Cal-I group and the Veh group acquired the SWSh task (Figure 4A). There was no main effect for group F(1,18) = 1.203, *p =* 0.248 and no interaction effect F(7,126) = 1.316, *p =* 0.248. Visual inspection of Figure 4A suggested a possible difference between our groups on Block 8 of acquisition training and this was tested using an independent samples *t-*test. The results revealed no significant difference between the groups T(18) = 1.508, *p =* 0.149 and since the Cal-I group was outperforming the Veh group, the study proceeded. Similar to the data for time to complete the task, a 2 (group) × 8 (training block) repeated measures ANOVA for within-phase errors revealed a significant main effect for training block F(7,126) = 2.92, *p =* 0.007, a marginal effect for group F(1,18) = 3.397, *p =* 0.082, and no interaction effect F(7,126) = 1.438, *p =* 0.196. For across-phase errors there was once again a main effect for training block F(7,126) = 3.072, *p =* 0.005, no main effect for group F(1,18) = 0.549, *p =* 0.468, and no interaction effect F(7,126) = 1.765, *p =* 0.10. Importantly, there was no significant difference between the Cal-I and Veh group on Block 8 for either across-phase errors T(18) = 1.454, *p =* 0.162 or within-phase errors T(18) = −0.599, *p =* 0.557.

Following the acquisition phase, animals underwent surgery. After recovery, six additional days of training were conducted post-surgery. These training days were averaged into three-day blocks. Cal-I and Veh groups showed no significant differences for time on either block 1 (T(14) = 1.581, *p =* 0.136) or block 2 (T(14) = 0.249, *p =* 0.807). Similarly, there were no differences for within-phase errors on either block 1 (T(14) = 1.196, *p =* 0.252) or block 2 (T(14) = 0.579, *p =* 0.572; see Figure 4B). Finally, no difference between the groups was seen for across-phase errors on block 1 (T(14) = 1.112, *p =* 0.285). However, significant differences were observed for block 2 (T(14) = 2.293, *p =* 0.038) with the Cal-I group committing significantly more across-phase errors than vehicle-injected controls.

### 3.4. Chronic Calpain Inhibition Blocks the Training-Induced Reduction in Cortactin

**Figure 5:**
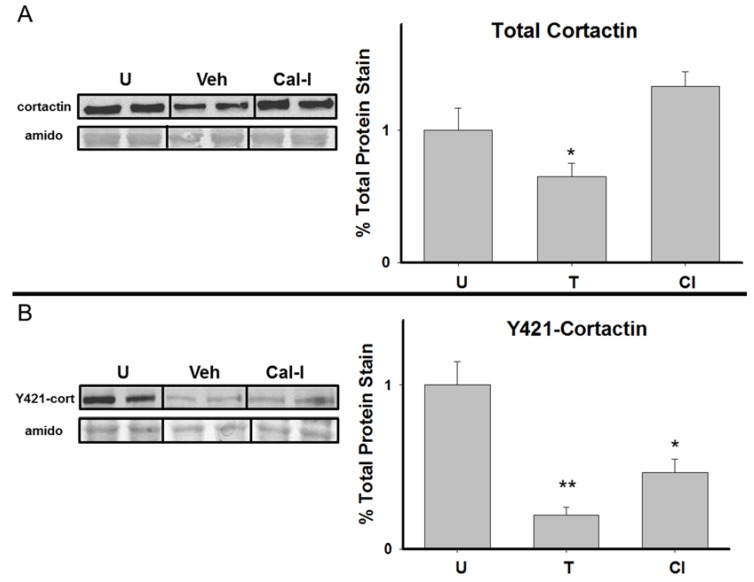
Chronic calpain inhibition blocks the reduction in total, but not Y421-phosphorylated cortactin following spatial working memory formation in the RAM. Representative samples and graphical depictions of group means for hippocampal total, and Y421-phosphorylated, cortactin (±SEM; expressed as percent of untrained controls) at 30 min following RAM training for Veh and Cal-I groups. * *p* < 0.05 compared to untrained controls, ** *p* < 0.05 compared to both untrained control and Cal-I groups.

Figure 5 shows a representative immunoblot and graphical representations of hippocampal cortactin and Y421-phosphorylated cortactin expression in untrained, Veh, and Cal- I rats. Due to limitations in the number of wells on a single electrophoresis gel (10 wells per gel), only two groups (*N* = 4/group) could be analyzed on a gel at a time. Because of this, protein levels from each of the groups were compared to each other using a series of *T-*tests. For illustrative purposes, Figure 5 includes images of gels that were run *N* = 2 per group to depict group differences visually on a single electrophoresis gel. Furthermore, the graphical representation in Figure 5 consists of data that was collapsed across gels (*N =* 4/group) by normalizing total protein stain values from the untrained group. This was done to simplify visual depiction of our results; however, because normalization can introduce error, statistical comparisons were made only within each individual gel, thus comparing only two groups at a time. Results for total cortactin levels revealed a significant reduction in the Veh group compared to untrained controls T(6) = 2.832, *p =* 0.03; thus replicating our results from experiment 1. Further, our data demonstrate significantly higher cortactin expression in Cal-I rats compared to the Veh group T(6) = 4.498, *p =* 0.004. Taken together, these findings indicate that chronic calpain inhibition blocks the training-induced reduction in cortactin following spatial learning in the RAM. Results for Y421-phosphorylated cortactin showed a significant difference between untrained and Veh groups with the Veh group showing a reduction T(6) = 6.324, *p =* 0.001, again replicating our results from experiment 1. However, we also observed significant differences between untrained and Cal-I groups T(6) = 3.245, *p =* 0.018 and between the Veh and Cal-I groups T(6) = −2.641, *p =* 0.038. This indicates that calpain inhibition significantly attenuates the reduction in Y421-phosphorylated cortactin following RAM training, but Y421-phosphorylated cortactin is still significantly reduced in the presence of calpain inhibitors.

## 4. Conclusions

Our main finding from the first experiment is that both total cortactin and Y421- phosphorylated cortactin are reduced 30 min post-training in the RAM. The magnitude of the reduction in total cortactin we observed is consistent with a previous report which demonstrated reduced hippocampal cortactin levels following spatial memory training in the water maze [19]. As in the previous study, our data demonstrate a reliable reduction in hippocampal cortactin following a spatial memory event. In addition, the present study provides novel data which show a comparable reduction in Y421-phosphorylated cortactin following spatial memory formation. The time course for the reduction in both total and Y421-phosphorylated cortactin is short-lived, with significant reductions seen at 30 min but not at 5 min or 4 h post-training. This timeline is somewhat earlier than that reported by Meighan and colleagues who saw significant reductions in total cortactin as late as 4 h after training [19]. The timeline for Y421-phosphorylated cortactin is consistent with other reports of reduced cortactin phosphorylation following learning [36].

To date, the data connecting cortactin and hippocampus-dependent memory performance are correlational in nature. It would be desirable to directly manipulate the functionality of cortactin in order to provide additional insight into its potential importance for learning and memory function; however, specific antagonists for cortactin that are suitable for *in vivo* injection have not yet been identified and genetic knockout of the protein has proven to be lethal to embryonic development. Given these constraints, we wondered what the potential outcome would be if we inhibited calpain, a known protease of cortactin in an attempt to block the reduction in cortactin that we observed in our first experiment. Therefore, our second experiment tested the effect of chronic calpain inhibition on total and Y421-phosphorylated hippocampal cortactin levels and on spatial working memory performance in the RAM. Our rationale was that if calpain inhibition blocked the training-induced reduction in cortactin and also impaired spatial working memory, it would provide further support for the notion that a reduction in hippocampal cortactin is important for optimal memory formation. Behaviorally, chronic calpain inhibition led to a deficit in spatial working memory performance in the RAM. While we cannot rule out the possibility that calpain inhibitors could impair RAM performance by affecting olfactory acuity rather than spatial memory, we believe this is unlikely due to the fact that our injections were given icv and that our results are consistent with previously published studies which show that calpain inhibitors impair spatial learning and interfere with LTP [24,27]. Specifically, our findings demonstrate that, while neither the latency to complete the task nor within-phase errors were significantly elevated as a result of calpain inhibition, there was a significant increase in across-phase errors in the calpain inhibitor group. This distinction is significant because across-phase errors measure delay-dependent spatial memories (where the animal was during a training session in the past), whereas within-phase errors assess delay-independent memories (where the animal has been in the current training session [30]). The finding that calpain inhibition impaired across-phase, but not within-phase spatial working memory performance makes sense from the standpoint that the longer the memory challenge, the more likely it is to require structural modifications within the hippocampus.

Our second experiment also showed that chronic calpain inhibition, in addition to impairing spatial working memory, significantly affected hippocampal cortactin levels. Similar to experiment 1, we observed a training-induced reduction in total and Y421-phosphorylated cortactin in the hippocampus 30 min post-training in the RAM. However, chronic icv administration of calpain inhibitors blocked the reduction in total and, to some degree, Y421-phosphorylated hippocampal cortactin 30 min post-training in the RAM. Taken together, we demonstrate that chronic calpain inhibition not only impairs spatial working memory performance, but it also blocks the reduction in hippocampal cortactin that occurs 30 min post-training in the RAM. While far from definitive on the exact nature of its involvement, our data advance understanding of the potential importance of cortactin for hippocampus-dependent memory; indeed, based on our results, it is tempting to speculate that a reduction in hippocampal cortactin is not only associated with, but may somehow be necessary for, hippocampus-dependent memory formation.

As mentioned previously, cortactin is an important regulator of the actin cytoskeleton, and cytoskeletal reorganization is critical for maintaining the ongoing stability of memories. Tyrosine phosphorylation of cortactin serves to translocate cortactin from dendritic spines to the shaft [37]; however, the result of this translocation and its potential importance in memory formation is poorly understood. It is possible that the hippocampus-wide reduction in cortactin we saw was because the translocation of cortactin led to its degradation. Support for this hypothesis can be derived from the finding that, in non-neuronal cells, tyrosine phosphorylation leads to cortactin degradation via calpain [38]. Therefore, degradation by calpain could be responsible for the widespread reduction in hippocampal cortactin we observed. Indirect support for this supposition can be taken from *in vitro* studies of cultured hippocampal neurons. Glutamate signaling via the NMDA receptor has been shown to increase cortactin phosphorylation on Y421 [37] and induce the translocation of cortactin away from dendritic spines [12]. NMDA receptor signaling also leads to the activation of calpain [22]. Additionally, during neuronal development, there is strong evidence that one of the major functions of calpains in hippocampal neurons is to concentrate in the dendritic shaft and limit cortactin levels in order to repress actin polymerization and thereby consolidate neurites [39], a finding which is also supported by non-neuronal cell culture work [21]. The idea that a calpain/cortactin relationship may exist during the stabilization of persistent forms of hippocampus-dependent memory is supported by the results of the present study. It is important to note, however, that calpain regulates a number of proteins and kinases critical for LTP induction and synaptic plasticity, including α-amino-3-hydroxy-5-methyl-4-isoxazoleproprionic acid (AMPA) and NMDA receptor subunits, second messengers, cytoskeleton-associated proteins, and transcription factors [25,27,40]. Therefore, we hasten to make the obvious statement that our data do not provide a direct demonstration of a role for calpain-mediated cortactin cleavage in hippocampus-dependent memory formation. In order to clarify this question, future studies should use immunohistology at various post-training time points in order to assess the neuronal distribution and potential co-localization of cortactin and calpain after spatial memory formation. Additionally, if the reduction in cortactin is due to degradation, it is reasonable to expect that the subsequent elevation in the protein is due to new cortactin protein synthesis. Therefore, quantification of cortactin mRNA following spatial working memory training would yield important additional information regarding the mechanism behind spatial memory-induced changes in hippocampal cortactin. Regardless, we have demonstrated that chronic calpain inhibitors, whether through directly inhibiting calpain-mediated cortactin degradation or through interfering with calpain’s effects on upstream targets, block the reduction in cortactin following spatial learning and impair spatial working memory in the RAM.

Future studies should target upstream regulators of cortactin in order to further elucidate the role and potential importance of this protein in synaptic plasticity and memory formation. Specifically, it will be important to test the effects of NMDA receptor ligands on our molecular targets because this would further elucidate whether NMDA signaling in the hippocampus is responsible for driving changes in both cortactin and Y421-phosphorylated cortactin levels. Other potential molecular targets which could further elucidate the role of cortactin in memory formation include inhibitors of the Src- and Abl-family kinases. Both families of kinases are known to directly phosphorylate cortactin on a number of tyrosine sites, an event which regulates the activity state of cortactin. In a recent study, Gourley and colleagues demonstrated that Abl family kinases in the dorsomedial striatum facilitate response-outcome learning in mice, likely through their interaction with cortactin [36]. Investigation of the relationship between Abl- and Src-family kinases and cortactin may shed additional light on the mechanisms underlying dendritic changes that appear to subserve hippocampus-dependent memory formation.

The present results support and add to previous work which demonstrates that changes in hippocampal cortactin are a correlate of spatial memory formation with likely importance for ongoing memory stability. Further, the present study demonstrates that calpain inhibition alters both spatial memory performance and hippocampal cortactin changes. These findings have potential importance for understanding psychological and neurological diseases with symptomatic declarative memory impairments.
